# A RNAi-based therapeutic *proof of concept* targets salmonid whirling disease *in vivo*

**DOI:** 10.1371/journal.pone.0178687

**Published:** 2017-06-02

**Authors:** Subhodeep Sarker, Simon Menanteau-Ledouble, Mohamed H. Kotob, Mansour El-Matbouli

**Affiliations:** 1 Clinical Division of Fish Medicine, Department for Farm Animals and Veterinary Public Health, University of Veterinary Medicine Vienna, Vienna, Austria; 2 Discipline of Pharmacology, School of Medical Sciences, Sydney Medical School, The University of Sydney, Sydney, New South Wales, Australia; 3 Department of Pathology, Faculty of Veterinary Medicine, Assiut University, Asyut, Egypt; Universidad Nacional Autónoma de México, MEXICO

## Abstract

*Myxobolus cerebralis* is a cnidarian-myxozoan parasite that causes salmonid whirling disease. *M*. *cerebralis* alternates between two hosts: (1) a vertebrate salmonid and (2) an invertebrate oligochaete, *Tubifex tubifex*. There is no successful treatment for salmonid whirling disease. MyxSP-1 is a *M*. *cerebralis* serine protease implicated in whirling disease pathogenesis. We hypothesized that short-interfering RNA (siRNA)-induced RNA interference (RNAi) can silence *MyxSP-1* in the invertebrate host and abrogate the *M*. *cerebralis* life cycle. This would preclude whirling disease infection in the salmonid host. To test this hypothesis, we first developed a siRNA delivery protocol in *T*. *tubifex*. Second, we determined the effective dose for siRNA treatment of *M*. *cerebralis*-infected *T*. *tubifex*. *M*. *cerebralis*-infected *T*. *tubifex* were treated with different concentrations of *MyxSP-1* or negative control siRNAs (1μM, 2μM, 5μM or 7μM) at 15°C for 24h, 48h, 72h and 96h, respectively. We monitored *MyxSP-1* knockdown using real-time quantitative PCR (qPCR). siRNA treatment with *MyxSP-1* siRNA at 2μM concentration for 24h at 15°C showed maximum significant *MyxSP-1* knockdown in *T*. *tubifex*. Third, we determined the time points in the *M*. *cerebralis* life cycle in *T*. *tubifex* at which siRNA treatment was most effective. *M*. *cerebralis-*infected *T*. *tubifex* were treated with *MyxSP-1* or negative control siRNAs (2μM concentration for 24h at 15°C) at 24 hours post-infection (24hpi), 48hpi, 72hpi, 96hpi, 1 month post-infection (1mpi), 2mpi and 3mpi, respectively. We observed that siRNA treatment of *T*. *tubifex* was most effective at 1mpi, 2mpi and 3mpi. Fourth, we immersed specific-pathogen-free rainbow trout fry in water inhabited by *MyxSP-1* siRNA-treated *T*. *tubifex* (at 1mpi, 2mpi and 3mpi). The salmonids did not develop whirling disease and showed significant *MyxSP-1* knockdown. We also observed long-term RNAi in *T*. *tubifex*. Together these results demonstrate a novel RNAi-based therapeutic *proof of concept in vivo* against salmonid whirling disease.

## Introduction

*Myxobolus cerebralis* is a cnidarian-myxozoan parasite and causative agent of whirling disease, an ecologically and economically debilitating disease of salmonids [1,2,3]. Salmonid whirling disease was first described in Germany in 1898 [1] and remains a disease of increasing importance. The disease was responsible for the significant decrease in rainbow trout (*Oncorhynchus mykiss*) populations in North America since the 1950s and was further exacerbated by a major outbreak in the mid-1990s [4]. Alarmed by this menace, the United States Congress awarded funding for the period between 1997 and 2006 to two organizations instituted for research associated with salmonid whirling disease *viz*., the Whirling Disease Foundation and the Whirling Disease Initiative through a cooperative agreement with the U.S. Fish and Wildlife Service [5]. Approximately US$8 million in U.S. federal funding was awarded during this period leading to the completion of >100 research studies through the Whirling Disease Initiative [5]. Despite the significant advances provided by the research investment, salmonid whirling disease continues to pose a severe threat to both the survival of rainbow trout in the wild and to aquaculture. For example, in 2016, the Canadian Food Inspection Agency (CFIA; Government of Canada) confirmed the first cases of salmonid whirling disease in Canada in 10 locations including the Banff National Park, Alberta [6]. This prompted the Alberta Environment and Parks to issue a Ministerial Fish Quarantine Order under section 32(2) of the *Fisheries (Alberta) Act* on September 6, 2016 for all commercial fish culture operations in the region [7].

Salmonid whirling disease is characterized by the following classical clinical symptoms: an eponymous tail-chasing whirling swimming behavior, blackening of the caudal region of the fish, shortening of the gill operculum and severe deformation of the vertebral column [1]. *M*. *cerebralis* has a complex two-host life cycle alternating between two obligate hosts: a vertebrate salmonid fish and an invertebrate oligochaete, *Tubifex tubifex*. The two-host life cycle of *M*. *cerebralis* is well described [8]. *M*. *cerebralis* alternates between two transmission stages: triactinomyxon (TAM) actinospores that develop in the invertebrate host and myxospores that develop in the salmonid host. *T*. *tubifex* is the only oligochaete species susceptible to *M*. *cerebralis*. The TAM stages of *M*. *cerebralis* are released into the water following a three-phase development in the intestinal epithelium of *T*. *tubifex*. TAMs are the only infective stages to the alternate salmonid hosts and penetrate the fish epidermis. The cells of the TAM sporoplasm undergo replication and dispersion in the epidermis and dermis before reaching the peripheral nerves and migrate to the cartilage. *M*. *cerebralis* is a tissue-specific parasite which primarily causes destruction of the salmonid host tissue cartilage. Several salmonid species are known as hosts of *M*. *cerebralis*. However, the rainbow trout *(Oncorhynchus mykiss)* is the most susceptible and exhibits the worst pathology [1]. Currently, there is no successful treatment for whirling disease-affected salmonid populations; culling remains the only viable strategy.

RNA interference (RNAi)-induced gene silencing of homologous mRNA transcripts by gene-specific exogenous double-stranded RNAs (dsRNAs) is an important tool to evaluate and validate new potential drug targets in human and veterinary medicine [9]. RNAi has been demonstrated in cells of mammals and other vertebrates including fish [10,11]. Since 2012, there has been a resurgence in clinical trials using RNA interference (RNAi)-based therapeutics for the treatment of diseases by specifically silencing the expression of disease-relevant genes [12]. Over 20 RNAi-based therapeutics are currently running clinical trials with several in Phase III trials [12]. RNAi has helped to explore the basic biology of debilitating parasites including *Schistosoma mansoni* in all stages of its life cycle [13]. Importantly, RNAi has been applied to silence gene expression in the intra-mammalian life stages (adults and schistosomula) of *S*. *mansoni* [13]. Gene silencing was evaluated by real-time quantitative PCR (qPCR) [13].

MyxSP-1 is a *M*. *cerebralis* serine protease belonging to the family of chymotrypsin-like serine proteases and has been implicated in whirling disease pathogenesis [14]. Based on the identification and initial characterization of *MyxSP-1* as a host-parasite interaction gene in salmonid whirling disease, we hypothesized that RNAi can knockdown *MyxSP-1* in the invertebrate host *in vivo*. This would abrogate the *M*. *cerebralis* life cycle in the invertebrate host and preclude whirling disease infection in the salmonid host. To test this hypothesis, we first developed a short-interfering RNA (siRNA) delivery protocol in *T*. *tubifex* and demonstrated potent and specific knockdown of *MyxSP-1* in *T*. *tubifex* [15]. Second, we determined the effective dose for siRNA treatment of *M*. *cerebralis*-infected *T*. *tubifex* at period of peak release of TAMs. Third, we determined the time points in the *M*. *cerebralis* life cycle in *T*. *tubifex* at which siRNA treatment of *M*. *cerebralis*-infected *T*. *tubifex* was most effective. Fourth, specific-pathogen-free rainbow trout fry when immersed in water inhabited by *MyxSP-1* siRNA-treated *T*. *tubifex* did not develop whirling disease and showed significant knockdown of *MyxSP-1*. Interestingly, we also observed siRNA-induced long-term RNAi in *T*. *tubifex*. Together these results demonstrate a novel RNAi-based therapeutic *proof of concept* that validates *MyxSP-1* as a potential new drug target in salmonid whirling disease.

## Materials and methods

### Culturing *T*. *tubifex* oligochaetes

Mud was collected from a commercial Austrian farm: Fischzucht Haimel, Kremserstrasse 65–67, 3133 Traismauer, Austria with no prior history of salmonid whirling disease. The mud was passed through a 1mm sieve net to remove coarse-grained particles. This refined mud was sterilized by heating in a drying oven at 150°C for 6h. The mud was then cooled and distributed in individual tanks (2L) up to a depth of about 5-8cm. A steady flow of tap water (pH 7±0.5, hardness 16.8°dH) was supplied overnight. A specific-pathogen-free (SPF) culture of *T*. *tubifex* oligochaetes were subsequently distributed on the mud layer of the individual tanks as shown previously [16,17]. The tanks were undisturbed for 2 days to allow the *T*. *tubifex* to burrow into the mud. Green algae wafers were crushed manually with a mortar and pestle and fed to the *T*. *tubifex*. The cultures were kept at a temperature of 15°C, stirred up weekly and light organics removed regularly.

### Enrichment of *M*. *cerebralis* myxospores and infection of *T*. *tubifex* oligochaetes

2-months old rainbow trout fry were obtained from the brood house of a commercial Austrian farm: Fischzucht Haimel, Kremserstrasse 65–67, 3133 Traismauer, Austria. They were experimentally infected with *M*. *cerebralis*. Fish showing clinical signs of whirling disease were used as source for collection of myxospores. Spore-bearing heads of diseased rainbow trout were immersed in water at ~40°C for 15 min to facilitate the separation of skeletal parts [16,17]. The skeletal parts were excised into ~1mm pieces and mechanically disrupted using Ultra Turrax^®^, then suspended in tap water and sequentially passed through screens with mesh sizes of 1000μm, 500μm, 250μm and 100μm, respectively. The final filtrate was centrifuged and the resulting pellets were resuspended in tap water. *M*. *cerebralis* myxospores were enumerated using a hemocytometer. The water flow was stopped in the 2L tanks with mud and *T*. *tubifex* and the enriched *M*. *cerebralis* spores were added at a dose of 400 myxospores/oligochaete [16,17]. After 24h, water flow was reinstated to a rate of 5-10ml.min^-1^ and water temperature maintained at 15°C.

### Design and synthesis of siRNAs and siRNA treatment of *T*. *tubifex* oligochaetes

siRNAs were designed using the siDirect v2.0 software (http://siDirect2.RNAi.jp/) [18]. Custom-designed siRNAs specific for *MyxSP-1* were obtained from Ambion, USA. The highest-ranking sense and antisense siRNA duplexes representing the best combination of activity and specificity were provided at 5 nM concentration as lyophilized power. The 5’-3’ sequences of the sense and antisense siRNA strands of *MyxSP-1* siRNA were: sense (GCUCAUUGCAUUGACUUUATT); antisense (UAAAGUCAAUGCAAUGAGCCC). In addition, two additional sets of siRNAs targeting *MyxSP-1* were designed and used *viz*., *MyxSP-1B* siRNA and *MyxSP-1C* siRNA. The 5’-3’ sequences of the sense and antisense strands of *MyxSP-1B* siRNA were: sense (GCGCUUUUCUGGCAUUUAATT); antisense (UUAAAUGCCAGAAAAGCGCAG). The 5’-3’ sequences of the sense and antisense strands of *MyxSP-1C* siRNA were: sense (CGAUUAGAAGAUGAAACUUTT); antisense (AAGUUUCAUCUUCUAAUCGUC). The 21bp Cy3-labelled negative control siRNA (#AM 4621, Ambion) does not have any sequence similarity to that of *T*. *tubifex*, *M*. *cerebralis*, *O*. *mykiss*, *Mus musculus*, *Rattus norvegicus* or *Homo sapiens* and does not target any gene products. It has been pretested (Ambion) in cell-based screens and proven to have no significant effect on cell proliferation, viability or morphology. As recommended by the manufacturer (Ambion), it was used as negative control siRNA to determine: (1) off-target effects, if any and (2) efficiency of siRNA delivery in *T*. *tubifex* as shown previously [15]. As per manufacturer’s instructions, separate stock solutions of 100μM were prepared for each set of siRNAs and stored at -20°C until use. We recently showed that soaking *T*. *tubifex* in RNAse-free water (Ambion) containing 2μM Cy3 labeled-negative controlled siRNA (#AM 4621, Ambion) in 1.5ml microcentrifuge tubes for 24 h at 15°C led to rapid uptake of the fluorescently labeled siRNA [15]. Fluorescence was detected using epifluorescence microscopy (Olympus) in the oligochaete intestine and hypodermis and intercellular pansporocysts of the parasite but not in the coelomic cavity [15]. We used this siRNA delivery protocol for determination of the effective dose for siRNA treatment in *T*. *tubifex*. siRNAs were used at a single dose without mixing at 1μM, 2μM, 5μM or 7μM final concentration for soaking *M*. *cerebralis*-infected *T*. *tubifex* at 15°C for 24h, 48h, 72h or 96h, respectively **(Table 1)**. *MyxSP-1* knockdown was subsequently evaluated post-soaking using real-time quantitative PCR (qPCR). For determination of the effective time point for siRNA treatment, *M*. *cerebralis*-infected *T*. *tubifex* were soaked in siRNAs (2μM concentration for 24h at 15°C) at 24 hours post-infection (24hpi), 48hpi, 72hpi, 96hpi, 1 month post-infection (1mpi), 2mpi and 3mpi **(Table 2)**. 24 hours after the final siRNA treatment at 3mpi, *MyxSP-1* knockdown was subsequently evaluated post-soaking for all time points using qPCR.

**Table 1:** Effective dose for siRNA treatment of *M*. *cerebralis*-infected *T*. *tubifex* oligochaetes at period of peak release of TAMs.

siRNAs	siRNA concentration (μM)	Duration of siRNA treatment (h)	Number of *T*. *tubifex* treated with siRNAs (n)	Normalized *MyxSP-1* gene expression (% ± S.E.)	% *MyxSP-1* knockdown
Negative Control siRNA	1	24	50	100 ± 4.08	12.81
*MyxSP-1* siRNA	1	24	50	87.19 ± 3.41	
Negative Control siRNA	2	24	50	100 ± 4.08	78.87
*MyxSP-1* siRNA	2	24	50	21.13 ± 2.89	
Negative Control siRNA	5	24	50	100 ± 4.08	21.67
*MyxSP-1* siRNA	5	24	50	78.33 ± 1.69	
Negative Control siRNA	7	24	50	100 ± 4.08	10.48
*MyxSP-1* siRNA	7	24	50	89.52 ± 1.93	
Negative Control siRNA	1	48	50	100 ± 4.08	1.53
*MyxSP-1* siRNA	1	48	50	98.47 ± 2.38	
Negative Control siRNA	2	48	50	100 ± 4.08	76.76
*MyxSP-1* siRNA	2	48	50	23.24 ± 4.74	
Negative Control siRNA	5	48	50	100 ± 4.08	0.67
*MyxSP-1* siRNA	5	48	50	99.33 ± 3.09	
Negative Control siRNA	7	48	50	100 ± 4.08	13.09
*MyxSP-1* siRNA	7	48	50	86.91 ± 2.79	
Negative Control siRNA	1	72	50	100 ± 1.84	3.5
*MyxSP-1* siRNA	1	72	50	96.5 ± 4.36	
Negative Control siRNA	2	72	50	100 ± 1.84	62.84
*MyxSP-1* siRNA	2	72	50	37.16 ± 1.09	
Negative Control siRNA	5	72	50	100 ± 1.84	1.06
*MyxSP-1* siRNA	5	72	50	98.94 ± 3.04	
Negative Control siRNA	7	72	50	100 ± 1.84	0.42
*MyxSP-1* siRNA	7	72	50	99.58 ± 4.94	
Negative Control siRNA	1	96	50	100 ± 1.84	6.95
*MyxSP-1* siRNA	1	96	50	93.05 ± 4.45	
Negative Control siRNA	2	96	50	100 ± 1.84	58.09
*MyxSP-1* siRNA	2	96	50	41.91 ± 1.87	
Negative Control siRNA	5	96	50	100 ± 1.84	7.84
*MyxSP-1* siRNA	5	96	50	92.16 ± 4.5	
Negative Control siRNA	7	96	50	100 ± 1.84	9.45
*MyxSP-1* siRNA	7	96	50	90.55 ± 4.7	

1600 SPF *T*. *tubifex* were collected and then divided into 32 groups with each having 50 SPF *T*. *tubifex* as indicated below. All groups of SPF *T*. *tubifex* were infected with *M*. *cerebralis* myxospores at the same time. At 3mpi, infected *T*. *tubifex* oligochaetes were treated with different concentrations of *MyxSP-1* siRNA or negative control siRNA (1μM, 2μM, 5μM or 7μM) at 15°C for 24h, 48h, 72h and 96h, respectively. Post-soaking, siRNA-treated *T*. *tubifex* were harvested and *MyxSP-1* gene expression was evaluated using qPCR. *MyxSP-1* gene expression was normalized to that of *M*. *cerebralis β-actin*. Data represent mean normalized expression (n = 6–8; +SE). Abbreviations: SPF = specific-pathogen-free; mpi = months post-infection; qPCR = real-time quantitative PCR.

**Table 2:** siRNA treatment of *M*. *cerebralis* infected-*T*. *tubifex* at different time points post-infection.

siRNAs	siRNA concentration (μM)	Time point of siRNA treatment of *T*. *tubifex* post-infection	Number of *T*. *tubifex* treated with siRNAs (n)	Normalized *MyxSP-1* gene expression (% ± S.E.)	% *MyxSP-1* knockdown
Negative Control siRNA	2	24hpi	50	100 ± 4.72	58.43
*MyxSP-1* siRNA	2	24hpi	50	41.57 ± 5.29	
Negative Control siRNA	2	48hpi	50	100 ± 4.72	58.83
*MyxSP-1* siRNA	2	48hpi	50	41.17 ± 4.4	
Negative Control siRNA	2	72hpi	50	100 ± 4.72	59.04
*MyxSP-1* siRNA	2	72hpi	50	40.96 ± 7.38	
Negative Control siRNA	2	96hpi	50	100 ± 4.72	58.8
*MyxSP-1* siRNA	2	96hpi	50	41.2 ± 6.08	
Negative Control siRNA	2	1mpi	50	100 ± 4.72	62.43
*MyxSP-1* siRNA	2	1mpi	50	37.57 ± 3.43	
Negative Control siRNA	2	2mpi	50	100 ± 4.72	75.13
*MyxSP-1* siRNA	2	2mpi	50	24.87 ± 4.39	
Negative Control siRNA	2	3mpi	50	100 ± 4.72	77.38
*MyxSP-1* siRNA	2	3mpi	50	22.62 ± 5.45	

700 SPF *T*. *tubifex* were collected and then divided into 14 groups with each having 50 SPF *T*. *tubifex* as indicated below. All groups of SPF *T*. *tubifex* were infected with *M*. *cerebralis* myxospores at the same time. Infected *T*. *tubifex* were treated with *MyxSP-1* siRNA or negative control siRNA (2μM concentration for 24h at 15°C) at 24hpi, 48hpi, 72hpi, 96hpi, 1mpi, 2mpi and 3mpi, respectively. 24 hours after the final siRNA treatment at 3mpi, siRNA-treated *T*. *tubifex* were harvested and *MyxSP-1* gene expression was evaluated using qPCR. *MyxSP-1* gene expression was normalized to that of *M*. *cerebralis β-actin*. Data represent mean normalized expression (n = 6–8; +SE). Abbreviations: SPF = specific-pathogen-free; hpi = hours post-infection; mpi = months post-infection; qPCR = real-time quantitative PCR.

### *In vivo* experiments using rainbow trout *(Oncorhynchus mykiss)* fry

All permits and approvals necessary for animal experiments in this study were obtained from the Austrian Federal Ministry of Science, Research & Economy (BMWFW) under the protocol number, “BMWFW-68.205/0045-WF/V/3b/2015”. All animal care and use institutional guidelines of the University of Veterinary Medicine Vienna and the BMWFW were followed. 180 specific-pathogen-free (SPF) rainbow trout *(Oncorhynchus mykiss)* fry were carefully chosen to represent the conditions in the farming environment. They were obtained as fry from the brood house of a commercial Austrian farm: Fischzucht Haimel, Kremserstrasse 65–67, 3133 Traismauer, Austria. The life-stage chosen was 2-months old rainbow trout fry because at this age the skeletal system predominantly comprises cartilage which favors the development of *M*. *cerebralis* in the salmonid host. All fish were obtained from the same batch and raised together under the same conditions until the beginning of the experiment to allow the results to be comparable between groups. *M*. *cerebralis*-infected *T*. *tubifex* were treated with negative control siRNA or *MyxSP-1* siRNA at 1 month post-infection (1mpi), 2mpi and 3mpi **(Table 3)**. 24 hours after the final siRNA treatment at 3mpi, 10 specific-pathogen-free rainbow trout fry in triplicates (ie. 3x10 = 30 fish) were immersed in water inhabited by negative control siRNA-treated *T*. *tubifex* (at 1mpi) at 15°C water without aeration for 30min. In addition, 10 specific-pathogen-free rainbow trout fry in triplicates (ie. 3x10 = 30 fish) were immersed in water inhabited by *MyxSP-1* siRNA-treated *T*. *tubifex* (at 1mpi) at 15°C water without aeration for 30min. Similarly, 10 specific-pathogen-free rainbow trout fry in triplicates (ie. 3x10 = 30 fish) were immersed in water inhabited by negative control siRNA-treated *T*. *tubifex* (at 2mpi) at 15°C water without aeration for 30min. In addition, 10 specific-pathogen-free rainbow trout fry in triplicates (ie. 3x10 = 30 fish) were immersed in water inhabited by *MyxSP-1* siRNA-treated *T*. *tubifex* (at 2mpi) at 15°C water without aeration for 30min. Finally, 10 specific-pathogen-free rainbow trout fry in triplicates (ie. 3x10 = 30 fish) were immersed in water inhabited by negative control siRNA-treated *T*. *tubifex* (at 3mpi) at 15°C water without aeration for 30min. Similarly, 10 specific-pathogen-free rainbow trout fry in triplicates (ie. 3x10 = 30 fish) were immersed in water inhabited by *MyxSP-1* siRNA-treated *T*. *tubifex* (at 3mpi) at 15°C water without aeration for 30min. 2h post-immersion, fish were transferred to separate 20L aquaria, each with 10 fish. The immersion procedure *per se* did not induce any mortalities. Each aquaria received 15°C well water at 2L.min^-1^ and fish were fed twice daily with a commercial trout diet. The fish were evaluated daily for development of clinical signs of salmonid whirling disease till the end of the experiment three months post-immersion. 3 months post-immersion, *T*. *tubifex* were harvested and the fish were euthanized with an overdose of the anesthetic, tricane methane-sulfonate (MS222) (400-500mg.l^-1^) [19]. Adequate steps were taken to minimize animal suffering. Prolonged immersion in this solution was used for euthanasia of moribund fish or prior to sampling. All sampling procedures were conducted post-mortem. Using sterile techniques, approximately 20-50mg of tissue from the cartilage was sampled from each fish and placed separately into sterile 1.5ml microcentrifuge tubes in RNA*later* and stored at -80°C till further use. *MyxSP-1* gene expression was evaluated using qPCR.

**Table 3:** Immersion of SPF rainbow trout fry in water inhabited by siRNA-treated *T*. *tubifex*.

SPF rainbow trout fry immersion groups (based on siRNA treatment of *M*. *cerebralis*-infected *T*. *tubifex*)	Time point of siRNA treatment of infected *T*. *tubifex* (months post-infection, mpi)	Number of SPF rainbow trout fry immersed in water inhabited by siRNA-treated *T*. *tubifex* (n)	Normalized *MyxSP-1* gene expression in rainbow trout fry (% ± S.E.)	% *MyxSP-1* knockdown in rainbow trout fry immersed in water inhabited by siRNA-treated *T*. *tubifex*	Normalized *MyxSP-1* gene expression in siRNA-treated *T*. *tubifex* at the end of the *in vivo* fish experiment (% ± S.E.)	% *MyxSP-1* knockdownin siRNA-treated *T*. *tubifex* at the end of the *in vivo* fish experiment
Neg. Ctrl. siRNA	1	30	100 ± 4.01	34.97	100 ± 4.07	61.69
*MyxSP-1* siRNA	1	30	65.03 ± 4.32		38.31 ± 3.32	
Neg. Ctrl. siRNA	2	30	100 ± 3.53	44.37	100 ± 3.73	72.37
*MyxSP-1* siRNA	2	30	55.63 ± 4.5		27.63 ± 4.1	
Neg. Ctrl. siRNA	3	30	100 ± 4.4	52.5	100 ± 4.9	77.46
*MyxSP-1* siRNA	3	30	47.5 ± 3.6		22.54 ± 3.3	

300 SPF *T*. *tubifex* were collected and then divided into 6 groups with each having 50 SPF *T*. *tubifex* as indicated below. All groups of SPF *T*. *tubifex* were infected with *M*. *cerebralis* myxospores at the same time. Infected *T*. *tubifex* were treated with *MyxSP-1* siRNA or negative control siRNA (2μM concentration for 24h at 15°C) at 1mpi, 2mpi and 3mpi, respectively. 24 hours after the final siRNA treatment at 3mpi, SPF rainbow trouts were immersed in water inhabited by *MyxSP-1* siRNA- or negative control siRNA-treated *T*. *tubifex [for details about immersion procedure please refer Materials and Methods section*: *In vivo experiments using rainbow trout (Oncorhynchus mykiss) fry]*. 3 months post-immersion, fish were euthanized and *T*. *tubifex* were harvested for *MyxSP-1* gene expression analyses using qPCR. *MyxSP-1* gene expression was normalized to that of *M*. *cerebralis β-actin*. Data represent mean normalized expression (n = 6–8; +SE). Abbreviations: SPF = specific-pathogen-free; mpi = months post-infection; qPCR = real-time quantitative PCR.

### RNA isolation, cDNA synthesis and real-time quantitative PCR (qPCR)

RNA was isolated from *T*. *tubifex* oligochaetes or rainbow trout cartilaginous tissue using the RNeasy Mini Kit (Qiagen) as per manufacturer’s instructions. Complementary DNA (cDNA) synthesis was done using 0.5μg RNA using the iScript cDNA synthesis kit (Bio-Rad) according to the manufacturer's instructions. The primers for *M*. *cerebralis β-actin (Mcbact)* and *MyxSP-1* were designed using sequence data from GenBank and are indicated in **(Table 4)**. The primers were tested for primer dimers and nonspecific amplicons, followed by optimization of individual annealing temperature and primer concentration for each primer pair. The target gene, predicted amplicon sizes and GenBank accession numbers are provided in **(Table 4)**. Gene expression was evaluated by real-time quantitative PCR (qPCR) using a CFX96^TM^ Real-Time System (Bio-Rad). To assess PCR efficiency, the PCR products were cloned using the TOPO TA Cloning Kit (Invitrogen) and tenfold dilution series in triplicates were used to generate the standard curve for each assay. The qPCR was performed in triplicate 20μl reaction volumes containing 1μg cDNA template, 0.5pmol forward and reverse primers for *M*. *cerebralis β-actin (Mcbact)* and *MyxSP-1*. Cycling parameters are shown in **(Table 5)**. Subsequent to each cycling protocol, a melting point curve followed, from 55°C every 30s for 0.5°C rising up to 95°C, to recognize nonspecific binding. The threshold cycles were taken from the annealing step. Efficiencies for the qPCR of the different genes were 86.9% for both *M*. *cerebralis β-actin (Mcbact)* and *MxySP-1*. The relative expression of the samples were calculated using the Bio-Rad CFX Manager v3.1 software for CFX96^TM^ Real-Time System (Bio-Rad) based on the algorithms outlined by Vandesompele et al (2002) [20]. *M*. *cerebralis β-actin (Mcbact)* was used as a reference gene for normalizing expression of target gene *MyxSP-1*. Transcript levels were measured as cycle threshold (*C*_t_) values and normalized to the housekeeping gene *M*. *cerebralis β-actin (Mcbact)*.

**Table 4:** Primers used in quantitative real-time PCR (qPCR).

Name	Amplicon size (bp)	Primer sequence (5’-3’)	GenBank accession no.
McbactF	160	CACCAGAAGAGCATCCCGTT	AY156508.2
McbactR		CCTGTAGTTCTTCCTGATGCGT	
MyxSP-1F	129	ACAATGCCAGGTGTGTCTGG	AY275708.2
MyxSP-1R		GGCTCCCTTTTCTAGTACGGAT	

**Table 5:** Thermal cycling parameters for quantitative real-time PCR (qPCR).

Target gene	Denature	Anneal	Elongate	Cycles
***β-actin (M*. *cerebralis)***	95°C/5min	-	-	1
	94°C/30s	55°C/30s	72°C/30s	35
***MyxSP-1 (M*. *cerebralis)***	95°C/5min	-	-	1
	94°C/30s	55°C/30s	72°C/30s	35

### Statistical analyses

The data were statistically analyzed using student paired *t*-tests. Analyses were done using GraphPad Prism v5 (San Diego, CA, USA). Values presented are mean normalized gene expression from six to eight independent qPCR assays + S.E for each siRNA treatment (^*^p<0.0001, ^**^p<0.001, ^***^p<0.005 and ^****^p<0.01 indicate statistical differences between *MyxSP-1* siRNA treatment and negative control siRNA treatment).

## Results

### Effective dose for siRNA treatment of *M*. *cerebralis*-infected *T*. *tubifex* oligochaetes at period of peak release of TAMs

**Fig 1:**
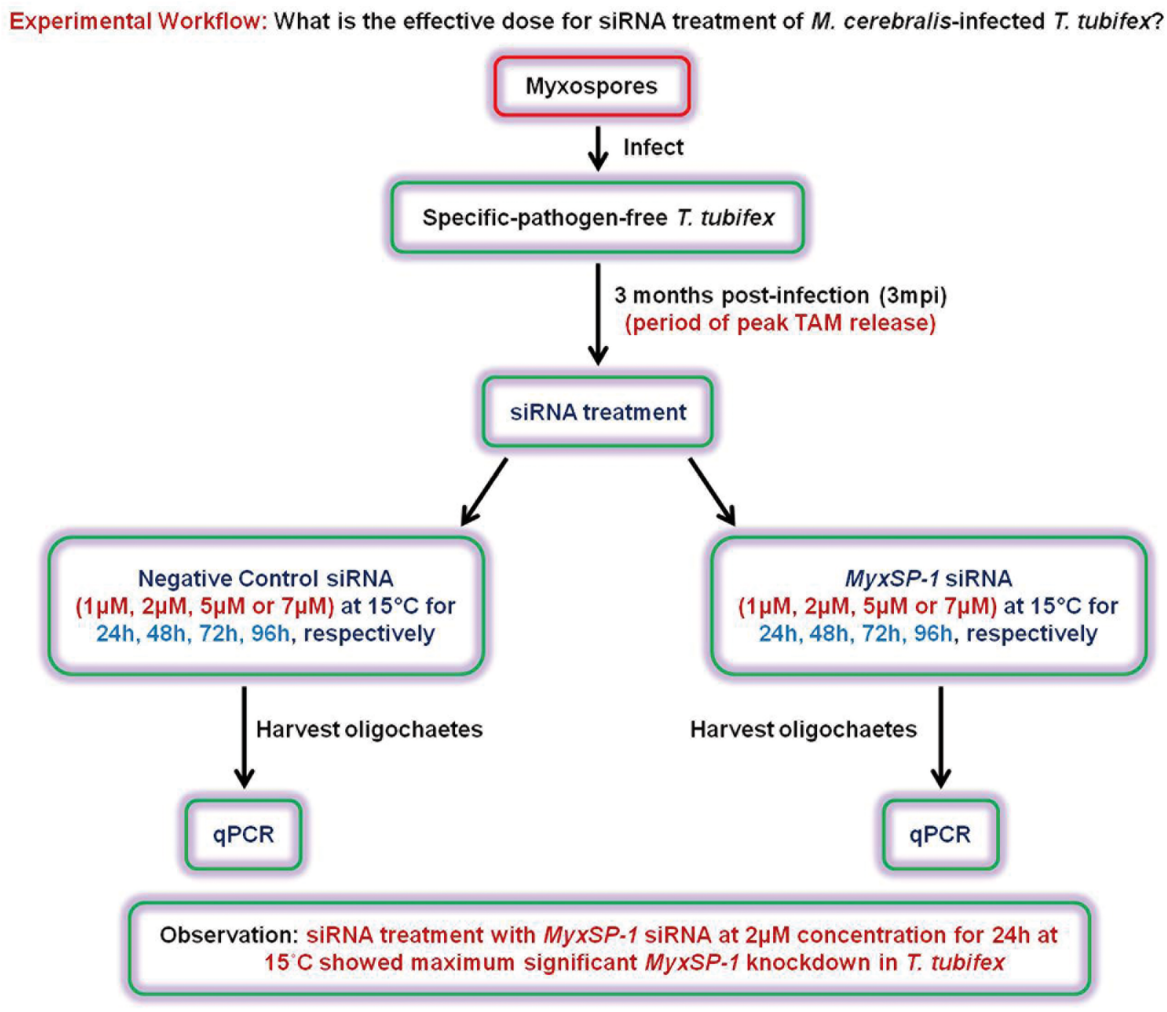
Schematic representation of the experimental workflow. What is the effective dose for siRNA treatment of *M*. *cerebralis*-infected *T*. *tubifex*?

**Fig 2:**
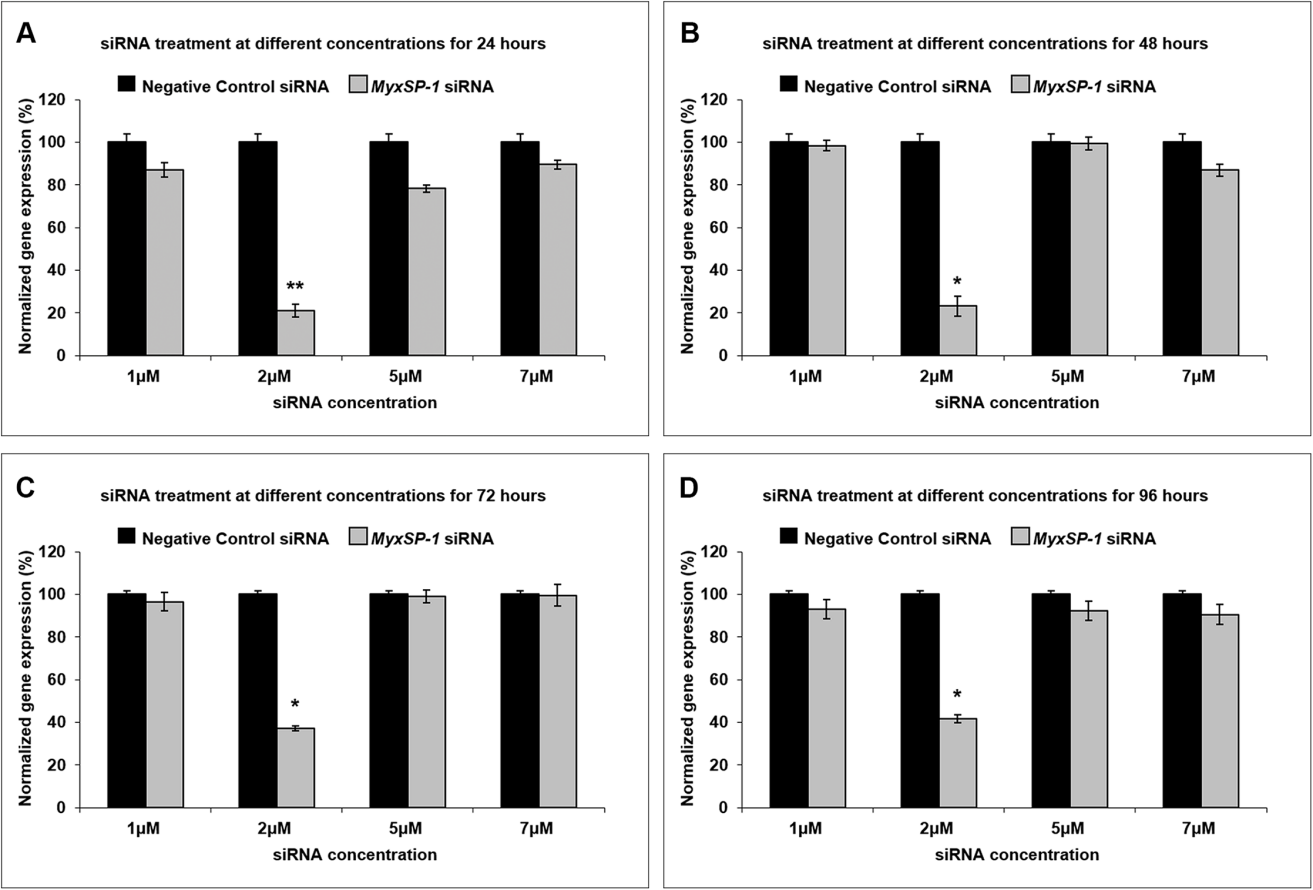
siRNA treatment of *T*. *tubifex* at different concentrations for 24h, 48h, 72h and 96h. 1600 SPF *T*. *tubifex* were collected and then divided into 32 groups with each having 50 SPF *T*. *tubifex*. All groups of SPF *T*. *tubifex* were infected with *M*. *cerebralis* myxospores at the same time. At 3mpi, infected *T*. *tubifex* oligochaetes were treated with different concentrations of *MyxSP-1* siRNA or negative control siRNA (1μM, 2μM, 5μM or 7μM, respectively) at 15°C for 24h **(A; n = 6–8; +SE;** ^**^p<0.001), 48h **(B; n = 6–8; +SE;** ^*^p<0.0001), 72h **(C; n = 6–8; +SE;** ^*^p<0.0001) and 96h **(D; n = 6–8; +SE;** ^*^p<0.0001). Post-soaking, siRNA-treated *T*. *tubifex* were harvested and *MyxSP-1* gene expression was evaluated using qPCR. *MyxSP-1* gene expression was normalized to that of *M*. *cerebralis β-actin*. Data represent mean normalized expression +SE. Abbreviations: SPF = specific-pathogen-free; mpi = months post-infection; qPCR = real-time quantitative PCR.

Specific-pathogen-free *T*. *tubifex* oligochaetes were infected with *M*. *cerebralis*. Infected *T*. *tubifex* show peak release of TAMs at 3 months post-infection (3mpi). Accordingly, at 3mpi, infected *T*. *tubifex* oligochaetes were treated with different concentrations of *MyxSP-1* siRNA or negative control siRNA (1μM, 2μM, 5μM or 7μM) at 15°C for 24h, 48h, 72h and 96h, respectively. We monitored the effect of siRNA treatment on *MyxSP-1* gene expression using real-time quantitative PCR (qPCR) after harvesting the oligochaetes **(Figs 1 and 2, Table 1)**. Similar data were obtained with two different sets of siRNAs targeting *MyxSP-1 viz*., *MyxSP-1B* siRNA and *MyxSP-1C* siRNA **(S1 and S2 Figs, S1 and S2 Tables)**. siRNA treatment with *MyxSP-1* siRNA at 2μM concentration for 24h at 15°C showed maximum significant knockdown, consistent with our previous observation [15]. For all subsequent siRNA experiments only *MyxSP-1* siRNA was used. This significantly reduced both: (i) the high cost of siRNA oligonucleotides and (ii) the required sample size for subsequent animal experiments using *T*. *tubifex* and rainbow trout.

### siRNA treatment of *M*. *cerebralis*-infected *T*. *tubifex* at different time points post-infection

**Fig 3:**
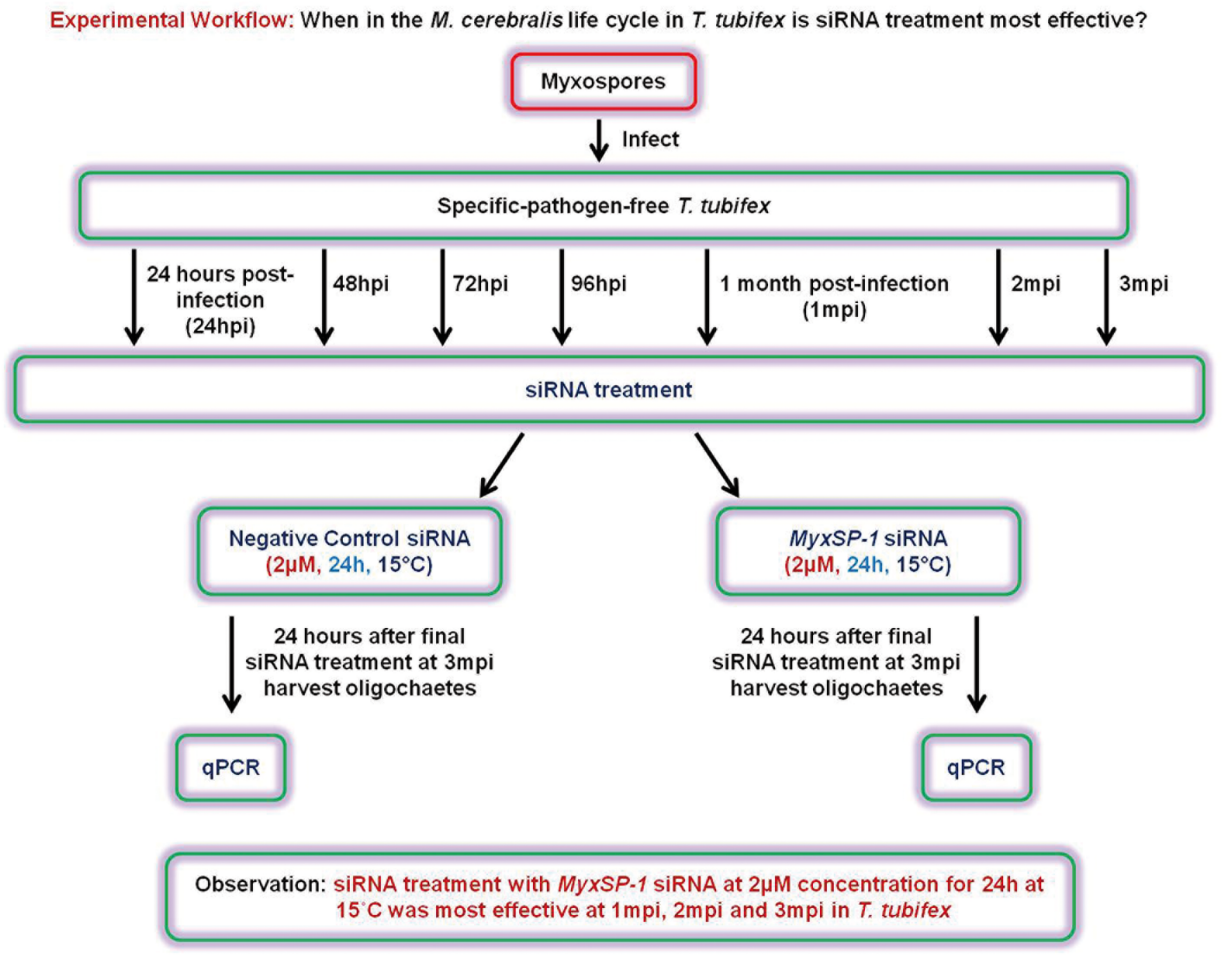
Schematic representation of the experimental workflow. When in the *M*. *cerebralis* life cycle in *T*. *tubifex* is siRNA treatment most effective?

**Fig 4:**
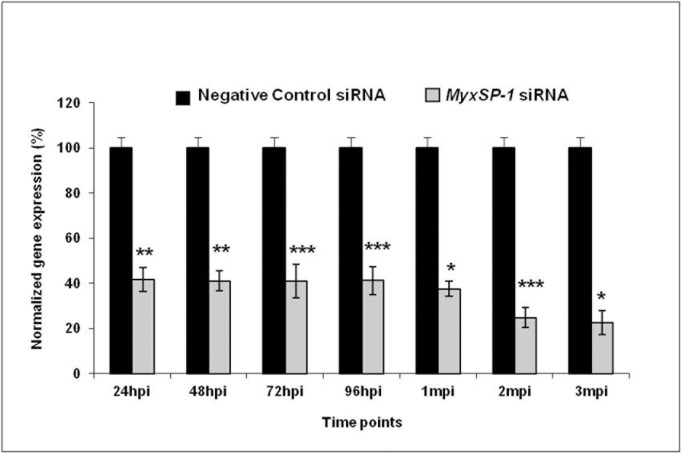
Normalized gene expression of *MyxSP-1* gene after siRNA treatment of *T*. *tubifex* at different time points post-infection. 700 SPF *T*. *tubifex* were collected and then divided into 14 groups with each having 50 SPF *T*. *tubifex*. All groups of SPF *T*. *tubifex* were infected with *M*. *cerebralis* myxospores at the same time. Infected *T*. *tubifex* were treated with *MyxSP-1* siRNA or negative control siRNA (2μM concentration for 24h at 15°C) at 24hpi, 48hpi, 72hpi, 96hpi, 1mpi, 2mpi and 3mpi, respectively. 24 hours after the final siRNA treatment at 3mpi, siRNA-treated *T*. *tubifex* were harvested and *MyxSP-1* gene expression was evaluated using qPCR. *MyxSP-1* gene expression was normalized to that of *M*. *cerebralis β-actin*. Data represent mean normalized expression (n = 6–8; +SE; ^*^p<0.0001; ^**^p<0.001; ^***^p<0.005). Abbreviations: SPF = specific-pathogen-free; hpi = hours post-infection; mpi = months post-infection; qPCR = real-time quantitative PCR.

Specific-pathogen-free *T*. *tubifex* oligochaetes were infected with *M*. *cerebralis* and treated with *MyxSP-1* siRNA or negative control siRNA (2μM concentration for 24h at 15°C) at 24 hours post-infection (24hpi), 48hpi, 72hpi, 96hpi, 1 month post-infection (1mpi), 2mpi and 3mpi, respectively. 24 hours after the final siRNA treatment at 3mpi, we harvested the oligochaetes in all treatment groups and monitored the effect of siRNA treatment on *MyxSP-1* gene expression in infected *T*. *tubifex* using qPCR **(Figs 3 and 4; Table 2)**. siRNA treatment of *M*. *cerebralis*-infected *T*. *tubifex* was most effective at 1mpi, 2mpi and 3mpi.

### *In vivo proof of concept*—siRNA treatment of *M*. *cerebralis*-infected *T*. *tubifex* precludes salmonid whirling disease in specific-pathogen-free rainbow trout fry

**Fig 5:**
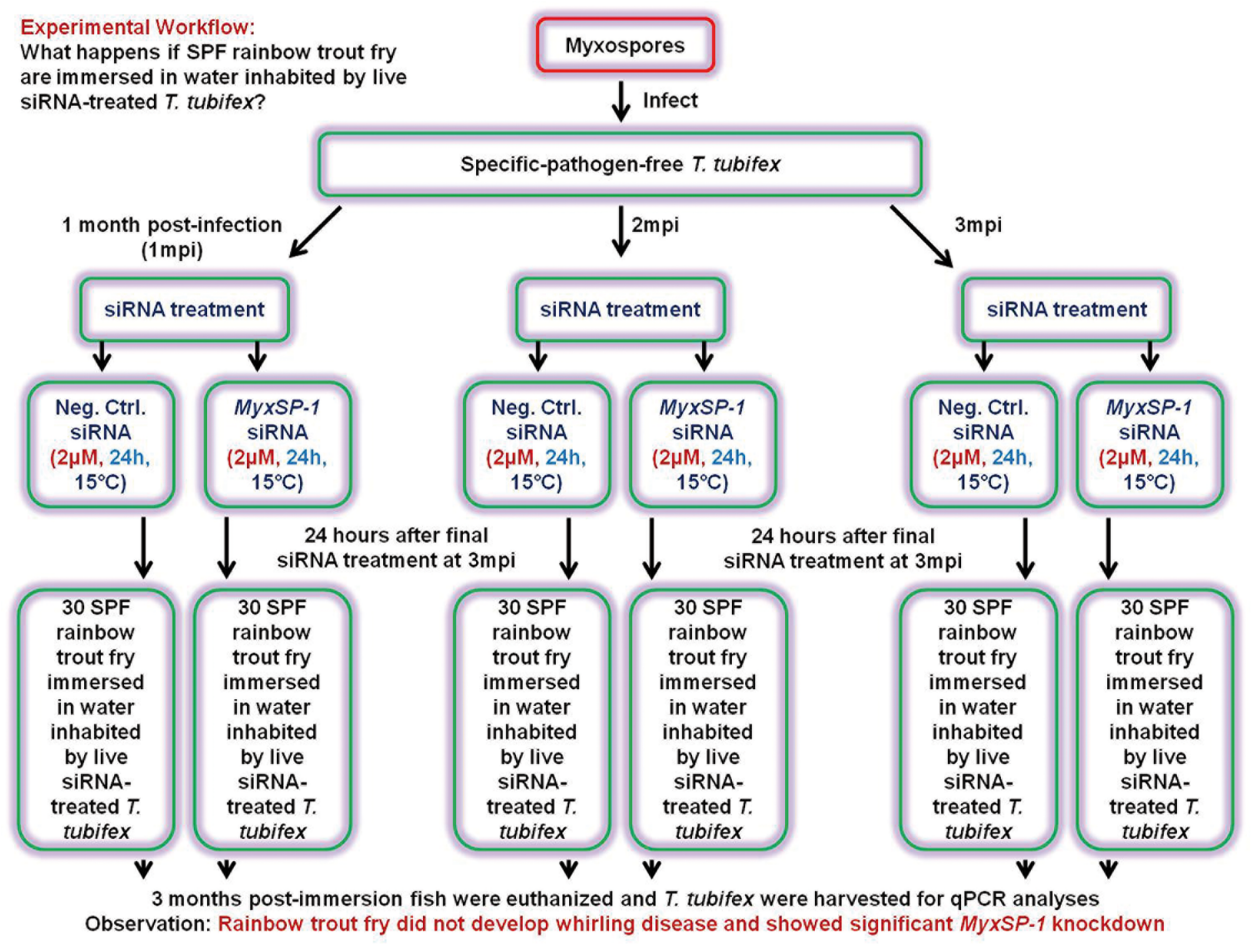
Schematic representation of the experimental workflow. What happens if SPF rainbow trout fry are immersed in water inhabited by live siRNA-treated *T*. *tubifex*?

**Fig 6:**
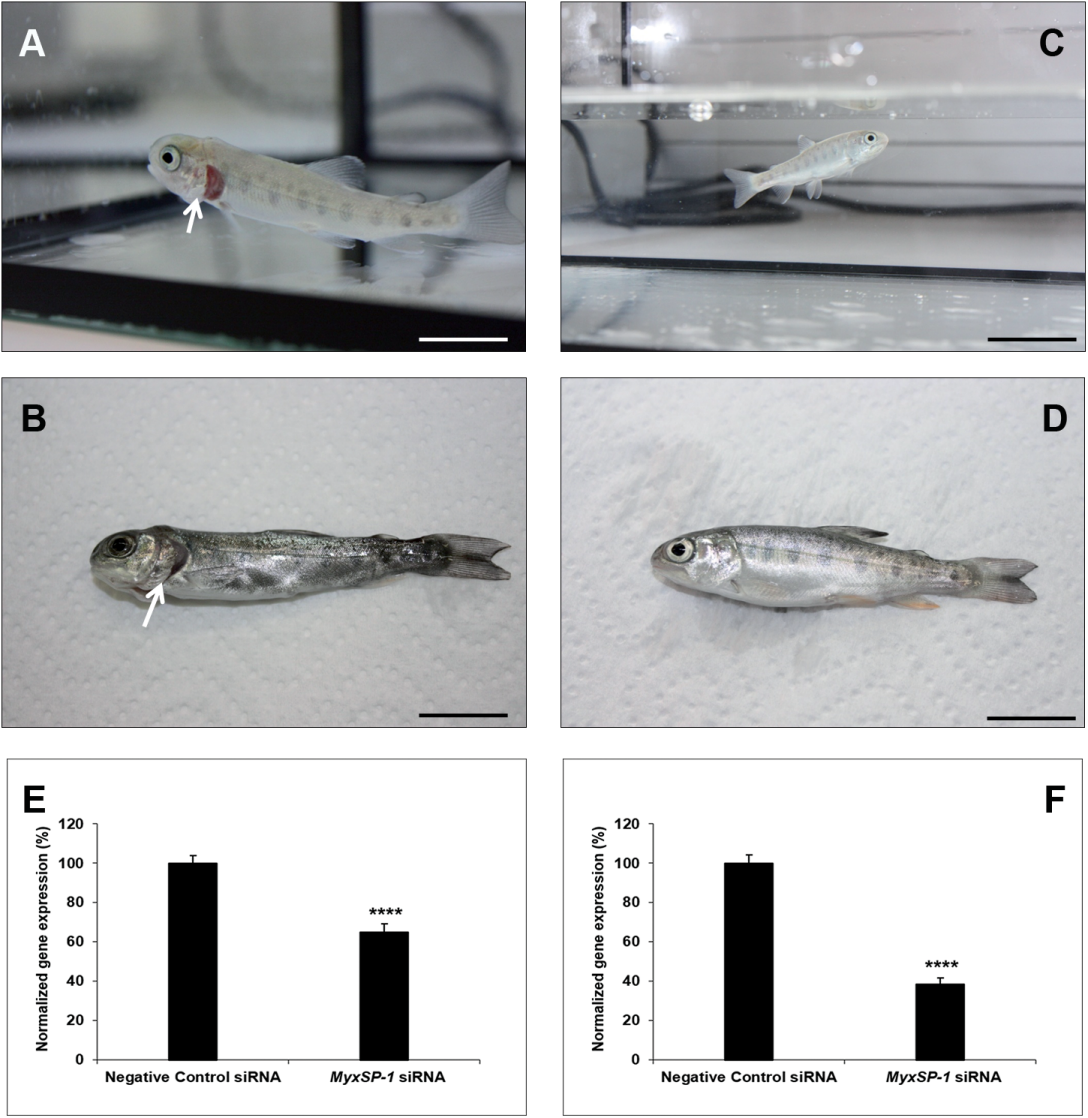
Immersion of SPF rainbow trout fry (n = 30; in triplicates of 10 fish per aquarium) in water inhabited by siRNA-treated *T*. *tubifex* 1 mpi. Representative image of rainbow trout fry photographed alive **(A, C)** and post-mortem **(B, D)** three months post-immersion in water inhabited by negative control siRNA-treated *T*. *tubifex* 1mpi **(A, B)** or *MyxSP-1* siRNA-treated *T*. *tubifex* 1mpi **(C, D)**. Arrow indicates clinical signs of whirling disease–shortening of the gill operculum **(A, B)**; scale bar = 2cm. 3 months post-immersion, fish were euthanized and *T*. *tubifex* were harvested. *MyxSP-1* gene expression was evaluated using qPCR. *MyxSP-1* gene expression was normalized to that of *M*. *cerebralis β-actin*. **(E)** Normalized gene expression of *MyxSP-1* in rainbow trout fry post-mortem. Data represent mean normalized expression (n = 6–8; +SE; ^****^p<0.01). **(F)** Normalized gene expression of *MyxSP-1* in siRNA-treated *T*. *tubifex* (1mpi) harvested at the end of the experiment. Data represent mean normalized expression (n = 6–8; +SE; ^****^p<0.01). Abbreviations: SPF = specific-pathogen-free; qPCR = real-time quantitative PCR.

**Fig 7:**
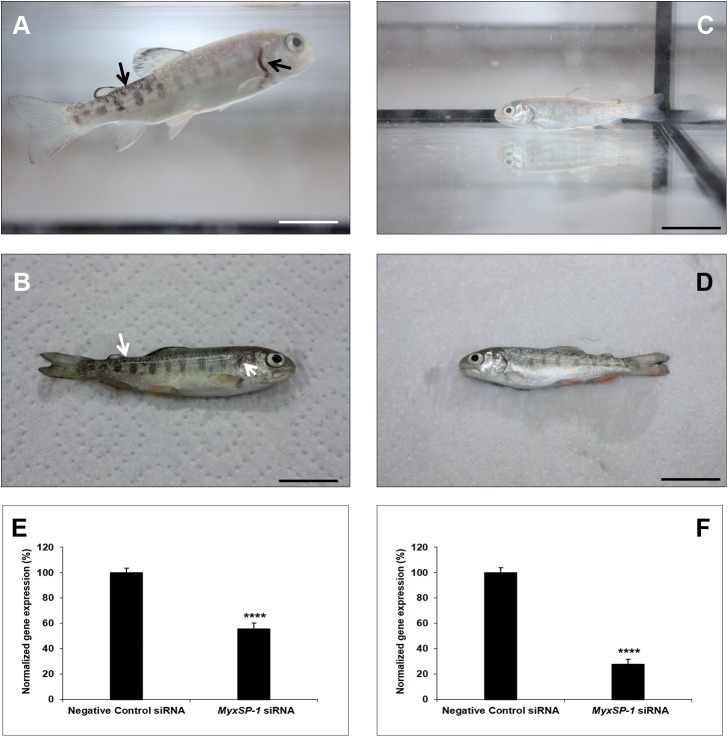
Immersion of SPF rainbow trout fry (n = 30; in triplicates of 10 fish per aquarium) in water inhabited by siRNA-treated *T*. *tubifex* 2mpi. Representative image of rainbow trout fry photographed alive **(A, C)** and post-mortem **(B, D)** three months post-immersion in water inhabited by negative control siRNA-treated *T*. *tubifex* 2mpi **(A, B)** or *MyxSP-1* siRNA-treated *T*. *tubifex* 2mpi **(C, D)**. Arrows indicate clinical signs of whirling disease–darkening of the caudal region and shortening of the gill operculum **(A, B)**; scale bar = 2cm. 3 months post-immersion, fish were euthanized and *T*. *tubifex* were harvested. *MyxSP-1* gene expression was evaluated using qPCR. *MyxSP-1* gene expression was normalized to that of *M*. *cerebralis β-actin*. **(E)** Normalized gene expression of *MyxSP-1* in rainbow trout fry post-mortem. Data represent mean normalized expression (n = 6–8; +SE; ^****^p<0.01). **(F)** Normalized gene expression of *MyxSP-1* in siRNA-treated *T*. *tubifex* (2mpi) harvested at the end of the experiment. Data represent mean normalized expression (n = 6–8; +SE; ^****^p<0.01). Abbreviations: SPF = specific-pathogen-free; qPCR = real-time quantitative PCR.

**Fig 8:**
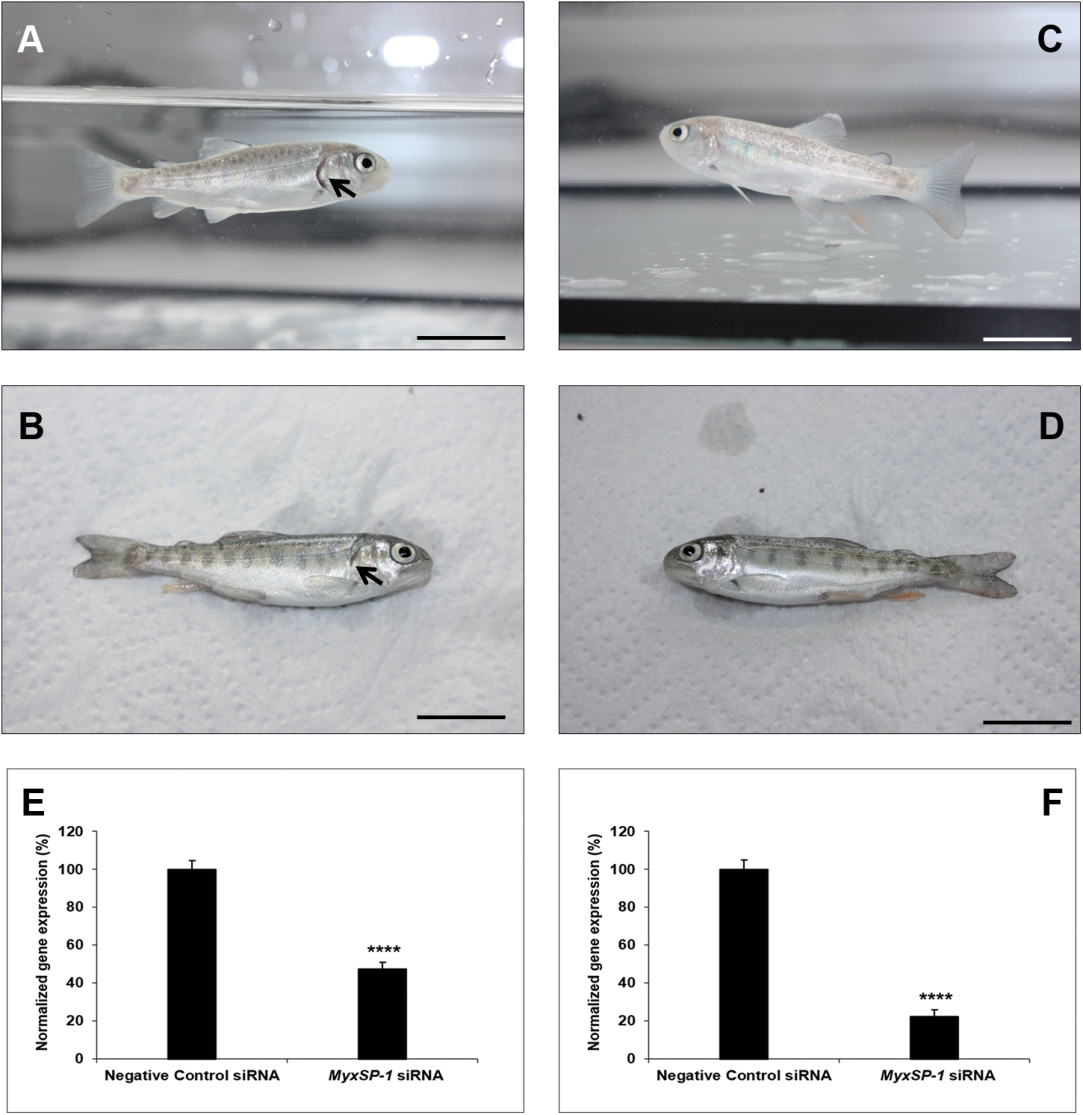
Immersion of SPF rainbow trout fry (n = 30; in triplicates of 10 fish per aquarium) in water inhabited by siRNA-treated *T*. *tubifex* 3mpi. Representative image of rainbow trout fry photographed alive **(A, C)** and post-mortem **(B, D)** three months post-immersion in water inhabited by negative control siRNA-treated *T*. *tubifex* 3mpi **(A, B)** or *MyxSP-1* siRNA-treated *T*. *tubifex* 3mpi **(C, D)**. Arrow indicates clinical signs of whirling disease–shortening of the gill operculum **(A, B)**; scale bar = 2cm. 3 months post-immersion, fish were euthanized and *T*. *tubifex* were harvested. *MyxSP-1* gene expression was evaluated using qPCR. *MyxSP-1* gene expression was normalized to that of *M*. *cerebralis β-actin*. **(E)** Normalized gene expression of *MyxSP-1* in rainbow trout fry post-mortem. Data represent mean normalized expression (n = 6–8; +SE; ^****^p<0.01). **(F)** Normalized gene expression of *MyxSP-1* in siRNA-treated *T*. *tubifex* (3mpi) harvested at the end of the experiment. Data represent mean normalized expression (n = 6–8; +SE; ^****^p<0.01). Abbreviations: SPF = specific-pathogen-free; qPCR = real-time quantitative PCR.

Specific-pathogen-free *T*. *tubifex* oligochaetes were infected with *M*. *cerebralis* and treated with *MyxSP-1* siRNA or negative control siRNA (2μM concentration for 24h at 15°C) at 1 month post-infection (1mpi), 2mpi and 3mpi, respectively. 24 hours after the final siRNA treatment at 3mpi, specific-pathogen-free rainbow trout fry were immersed in water inhabited by live siRNA-treated *T*. *tubifex*. The rainbow trout fry were regularly monitored for clinical signs of whirling disease for 3 months post-immersion. After 3 months post-immersion, the rainbow trout fry were euthanized and the siRNA-treated *T*. *tubifex* were harvested for gene expression analyses **(Fig 5; Table 3)**. We observed that rainbow trout fry immersed in water inhabited by negative control siRNA-treated *T*. *tubifex* (*M*. *cerebralis*-infected; 1mpi) showed clinical signs of salmonid whirling disease–shortening of the gill operculum **(Fig 6A and 6B)**. However, fish immersed in water inhabited by *MyxSP-1* siRNA-treated *T*. *tubifex* (*M*. *cerebralis*-infected; 1mpi) did not show clinical signs of salmonid whirling disease **(Fig 6C and 6D)** with ~35% knockdown in *MyxSP-1* gene expression in the cartilage determined by qPCR **(Fig 6E).** We also observed ~62% *MyxSP-1* knockdown in *MyxSP-1* siRNA-treated *T*. *tubifex* (*M*. *cerebralis*-infected; 1mpi) harvested at the end of the experiment **(Fig 6F)**. In addition, rainbow trout fry immersed in water inhabited by negative control siRNA-treated *T*. *tubifex* (*M*. *cerebralis*-infected; 2mpi) showed clinical signs of salmonid whirling disease–shortening of the gill operculum and darkening of the caudal region **(Fig 7A and 7B)**. In contrast, fish immersed in water inhabited by *MyxSP-1* siRNA-treated *T*. *tubifex* (*M*. *cerebralis*-infected; 2mpi) did not show clinical signs of salmonid whirling disease **(Fig 7C and 7D)** with ~44% knockdown in *MyxSP-1* gene expression in the cartilage determined by qPCR **(Fig 7E)**. We observed ~72% *MyxSP-1* knockdown in *MyxSP-1* siRNA-treated *T*. *tubifex* (*M*. *cerebralis*-infected; 2mpi) harvested at the end of the experiment **(Fig 7F)**. Finally, rainbow trout fry immersed in water inhabited by negative control siRNA-treated *T*. *tubifex* (*M*. *cerebralis*-infected; 3mpi) showed clinical signs of salmonid whirling disease–shortening of the gill operculum **(Fig 8A and 8B)**. But, fish immersed in water inhabited by *MyxSP-1* siRNA-treated *T*. *tubifex* (*M*. *cerebralis*-infected; 3mpi) did not show clinical signs of salmonid whirling disease **(Fig 8C and 8D)** with ~53% knockdown in *MyxSP-1* gene expression in the cartilage determined by qPCR **(Fig 8E)**. We observed ~77% *MyxSP-1* knockdown in *MyxSP-1* siRNA-treated *T*. *tubifex* (*M*. *cerebralis*-infected; 3mpi) harvested at the end of the experiment **(Fig 8F)**.

## Discussion

Some of the intervention strategies to manage salmonid whirling disease include temperature or chemical control [21], UV treatment of water [22] and habitat alteration [23]. Biological control of salmonid whirling disease through the establishment of *M*. *cerebralis*-resistant rainbow trout lineages has also been tested [24]. Quantitative genetic approaches and selective breeding programs have been used to alter population characteristics in several salmonid species [24,25]. Varying differences in salmonid susceptibility provide an opportunity to investigate the role of host factors in determining salmonid resistance to whirling disease [26]. Several studies have also identified genes in the salmonid host which are differentially expressed in whirling disease [27,28,29]. In addition, there are many studies that have investigated the genetic correlates of whirling disease in the oligochaete host [30–34]. However, most studies have concentrated either on the salmonid or the oligochaete hosts and largely overlooked the genetic correlates of whirling disease pathogenesis in *M*. *cerebralis* which is the causative agent. It is critical to understand *M*. *cerebralis* gene function in whirling disease pathogenesis for a more holistic approach to disease control.

One of the major challenges in the development of RNAi-based therapeutics for the specific inhibition of disease-relevant genes is the *in vivo* delivery of small interfering RNAs (siRNAs) [9–12]. For successful silencing of intracellular parasite genes, siRNAs must: (i) be systemically delivered from the area of uptake to body tissues harbouring and affected by the parasite, (ii) be taken up by the RNA-induced silencing complex and (iii) degrade specific messenger RNA targets of the parasite [10]. In whirling disease, this would suggest siRNA delivery to the rainbow trout cartilage because it is the specific tissue affected by *M*. *cerebralis*. However, the successful achievement of systemic siRNA delivery in invertebrates has been difficult to recapitulate in vertebrates and larger animals [35]. We addressed this challenge and developed an siRNA delivery approach to target *M*. *cerebralis* in its invertebrate host *(T*. *tubifex)* and not in its vertebrate host (rainbow trout). Since TAMs are the causative agent of whirling disease and they develop in *T*. *tubifex*, siRNA treatment in the invertebrate host would preclude whirling disease in rainbow trout. This was the underlying rationale for siRNA treatment of *T*. *tubifex*.

In order to determine the effective dose for siRNA treatment of *M*. *cerebralis*-infected *T*. *tubifex* oligochaetes, we administered siRNAs to high TAM-releasing oligochaetes **(Fig 1)**. We know that infected oligochaetes show peak release of TAMs at 3 months post-infection (3mpi) [16,17]. Hence, we used *T*. *tubifex* at 3mpi for determining the siRNA dose-response **(Fig 2)**. After establishing the effective dose for siRNA treatment of infected oligochaetes, we sought to determine the time points in the *M*. *cerebralis* life cycle in *T*. *tubifex* at which siRNA treatment would be most effective **(Fig 3)**. We observed significant *MyxSP-1* knockdown in *T*. *tubifex* at all time points post-infection **(Fig 4)**. However, *MyxSP-1* knockdown was most pronounced at 1mpi, 2mpi and 3mpi **(Fig 4)**. This is consistent with schizogony, gametogony and sporogony in the *M*. *cerebralis* life cycle in *T*. *tubifex* [8]. Schizogony occurs 0-1mpi in *T*. *tubifex* and involves multiple nuclear divisions of the binucleated sporoplasms derived from myxospores. These nuclear divisions produce numerous uninucleate cells which undergo further nuclear and cellular divisions as they migrate through the gut epithelia of infected *T*. *tubifex*. Gametogony occurs 1-2mpi in *T*. *tubifex* and is the only phase in the life cycle of *M*. *cerebralis* in which meiosis (sexual reproduction) occurs. Sporogony is the final stage of *M*. *cerebralis* development in *T*. *tubifex* which occurs 2-3mpi leading to a final pansporocyst harbouring 8 folded TAMs before release.

The process of filtering and concentrating TAMs from *M*. *cerebralis*-infected *T*. *tubifex* involves significant loss of actinospores due to several steps of filtration and concentration using a 20μm nitex screen [17]. This does not impede efficient infection of rainbow trout fry under laboratory conditions when dealing with millions of TAMs obtained from gram cultures of *M*. *cerebralis*-infected *T*. *tubifex*, typically required for laboratory maintenance of the *M*. *cerebralis* life cycle. However, siRNA treatment of gram cultures of *M*. *cerebralis*-infected *T*. *tubifex* would incur significant high costs for siRNA oligonucleotides. We know that each *M*. *cerebalis*-infected *T*. *tubifex* releases on average 8–10 TAMs at period of peak release, 3 mpi [17] and that an infection dose of ~400 TAMs/fish can optimally lead to whirling disease under laboratory conditions [16,17]. Hence, in this study, 50 infected *T*. *tubifex* were used in each experiment involving siRNA treatment (with negative control siRNA or *MyxSP-1* siRNA) **(Figs 2 and 4)**. To avoid loss of infective TAMs due to the filtration and concentration artifact including manual handling, we infected specific-pathogen-free rainbow trout fry by immersion in water inhabited by siRNA-treated *T*. *tubifex*. After siRNA treatment of *T*. *tubifex* and before fish immersion, we evaluated TAM infectivity but did not enumerate the number of infective TAMs due to the low TAM production rate (~400 TAMs produced by 50 *T*. *tubifex* in each siRNA treatment condition). Nevertheless, the absence of clinical symptoms of whirling disease in rainbow trout fry exposed to *MyxSP-1* siRNA-treated *T*. *tubifex* **(Figs 6–8)** suggests that *MyxSP-1* knockdown in *T*. *tubifex* during schizogony, gametogony and sporogony were critical to ensure loss of TAM infectivity for the fish. Our results show that *MyxSP-1* siRNA treatment of *T*. *tubifex* at 3mpi is most effective to target salmonid whirling disease *in vivo*.

The concomitant knockdown in *MyxSP-1* in the cartilage of rainbow trout fry exposed to *MySP-1* siRNA-treated *T*. *tubifex* **(Figs 6E, 7E and 8E)** is consistent with the fact that *MyxSP-1* is a crucial determinant for whirling disease pathogenesis in the rainbow trout. *MyxSP-1* transcription was previously detected in association with parasite developmental stages in cartilage associated with cartilage destruction and during lesion development. Interestingly, at the end of the current study, we also observed ~62% *MyxSP-1* knockdown in *MyxSP-1* siRNA-treated *T*. *tubifex* (*M*. *cerebralis*-infected; 1mpi) **(Fig 6F)**, ~72% *MyxSP-1* knockdown in *MyxSP-1* siRNA-treated *T*. *tubifex* (*M*. *cerebralis*-infected; 2mpi) **(Fig 7F)** and ~77% *MyxSP-1* knockdown in *MyxSP-1* siRNA-treated *T*. *tubifex* (*M*. *cerebralis*-infected; 3mpi) **(Fig 8F)**. This suggests induction of long-term RNAi in *T*. *tubifex* by a one-time single dose administration of siRNAs which is detectable at six months post-infection (6mpi). siRNA-induced long-term gene silencing was observed in an invertebrate a decade ago [36]. Vastenhouw et al. (2006) [36] showed that a single episode of RNAi in *C*. *elegans* can induce transcriptional gene silencing where the knockdown effect is inherited indefinitely even in the absence of the original RNAi trigger. This opens up the possibility to explore the biology of long-term RNAi in *T*. *tubifex* in more detail.

## Conclusions

*M*. *cerebralis* is an obligate parasite that cannot live independently of either host. Consequently, establishing a genetically tractable *in vitro* culture model of *M*. *cerebralis* outside either host remains elusive. We addressed this challenge and herein provided both an *in vitro* [15] and *in vivo* (the current study) *proof of concept* for molecular targeting of salmonid whirling disease using RNAi. First, we developed an efficient protocol to deliver *MyxSP-1* siRNA in *T*. *tubifex*. Second, we showed that siRNA treatment with *MyxSP-1* siRNA at 2μM concentration for 24h at 15°C showed maximum significant *MyxSP-1* knockdown in *T*. *tubifex*. Third, we observed that *MyxSP-1* siRNA treatment of *T*. *tubifex* was most effective at 1mpi, 2mpi and 3mpi. Finally, we showed that the salmonids did not develop whirling disease and showed significant *MyxSP-1* knockdown when immersed in water inhabited by *MyxSP-1* siRNA-treated *T*. *tubifex*. Interestingly, we also observed induction of long-term RNAi in *T*. *tubifex*. In conclusion, the strategy demonstrated in this paper may be of therapeutic relevance for diseases whose causative agent is an obligate parasite with a complex two-host life cycle. Genomic, transcriptomic and proteomic analyses of *M*. *cerebralis* pathogenesis would facilitate further target identification for silencing using RNAi-based therapeutics. The results presented here provide novel insights into *MyxSP-1* gene function in salmonid whirling disease.

## Supporting information

S1 Fig *MyxSP-1B* siRNA treatment of *T*. *tubifex* at different concentrations for 24h, 48h, 72h and 96h. 1600 SPF *T*. *tubifex* were collected and then divided into 32 groups with each having 50 SPF *T*. *tubifex*. All groups of SPF *T*. *tubifex* were infected with *M*. *cerebralis* myxospores at the same time. At 3mpi, infected *T*. *tubifex* oligochaetes were treated with different concentrations of *MyxSP-1B* siRNA or negative control siRNA (1μM, 2μM, 5μM or 7μM, respectively) at 15°C for 24h **(A; n = 6–8; +SE;** ^*^p<0.0001), 48h **(B; n = 6–8; +SE;** ^*^p<0.0001), 72h **(C; n = 6–8; +SE;** ^*^p<0.0001) and 96h **(D; n = 6–8; +SE;** ^*^p<0.0001). Post-soaking, siRNA-treated *T*. *tubifex* were harvested and *MyxSP-1* gene expression was evaluated using qPCR. *MyxSP-1* gene expression was normalized to that of *M*. *cerebralis β-actin*. Data represent mean normalized expression +SE. Abbreviations: SPF = specific-pathogen-free; mpi = months post-infection; qPCR = real-time quantitative PCR.
(TIF)

S2 Fig *MyxSP-1C* siRNA treatment of *T*. *tubifex* at different concentrations for 24h, 48h, 72h and 96h. 1600 SPF *T*. *tubifex* were collected and then divided into 32 groups with each having 50 SPF *T*. *tubifex*. All groups of SPF *T*. *tubifex* were infected with *M*. *cerebralis* myxospores at the same time. At 3mpi, infected *T*. *tubifex* oligochaetes were treated with different concentrations of *MyxSP-1C* siRNA or negative control siRNA (1μM, 2μM, 5μM or 7μM, respectively) at 15°C for 24h **(A; n = 6–8; +SE;** ^*^p<0.0001), 48h **(B; n = 6–8; +SE;** ^*^p<0.0001), 72h **(C; n = 6–8; +SE;** ^*^p<0.0001) and 96h **(D; n = 6–8; +SE;** ^*^p<0.0001). Post-soaking, siRNA-treated *T*. *tubifex* were harvested and *MyxSP-1* gene expression was evaluated using qPCR. *MyxSP-1* gene expression was normalized to that of *M*. *cerebralis β-actin*. Data represent mean normalized expression +SE. Abbreviations: SPF = specific-pathogen-free; mpi = months post-infection; qPCR = real-time quantitative PCR.
(TIF)

S1 Table Effective dose for *MyxSP-1B* siRNA treatment of *M*. *cerebralis*-infected *T*. *tubifex* oligochaetes at period of peak release of TAMs. 1600 SPF *T*. *tubifex* were collected and then divided into 32 groups with each having 50 SPF *T*. *tubifex* as indicated below. All groups of SPF *T*. *tubifex* were infected with *M*. *cerebralis* myxospores at the same time. At 3mpi, infected *T*. *tubifex* oligochaetes were treated with different concentrations of *MyxSP-1B* siRNA or negative control siRNA (1μM, 2μM, 5μM or 7μM) at 15°C for 24h, 48h, 72h and 96h, respectively. Post-soaking, siRNA-treated *T*. *tubifex* were harvested and *MyxSP-1* gene expression was evaluated using qPCR. *MyxSP-1* gene expression was normalized to that of *M*. *cerebralis β-actin*. Data represent mean normalized expression (n = 6–8; +SE). Abbreviations: SPF = specific-pathogen-free; mpi = months post-infection; qPCR = real-time quantitative PCR.
(DOCX)

S2 Table Effective dose for *MyxSP-1C* siRNA treatment of *M*. *cerebralis*-infected *T*. *tubifex* oligochaetes at period of peak release of TAMs. 1600 SPF *T*. *tubifex* were collected and then divided into 32 groups with each having 50 SPF *T*. *tubifex* as indicated below. All groups of SPF *T*. *tubifex* were infected with *M*. *cerebralis* myxospores at the same time. At 3mpi, infected *T*. *tubifex* oligochaetes were treated with different concentrations of *MyxSP-1C* siRNA or negative control siRNA (1μM, 2μM, 5μM or 7μM) at 15°C for 24h, 48h, 72h and 96h, respectively. Post-soaking, siRNA-treated *T*. *tubifex* were harvested and *MyxSP-1* gene expression was evaluated using qPCR. *MyxSP-1* gene expression was normalized to that of *M*. *cerebralis β-actin*. Data represent mean normalized expression (n = 6–8; +SE). Abbreviations: SPF = specific-pathogen-free; mpi = months post-infection; qPCR = real-time quantitative PCR.
(DOCX)
