# Supplementary figures and images for: A RNAi-based therapeutic proof of concept targets salmonid whirling disease in vivo
Source: PLoS One. 2017 Jun 2;12(6):e0178687. doi: 10.1371/journal.pone.0178687 (PMC5456292; doi:10.1371/journal.pone.0178687)

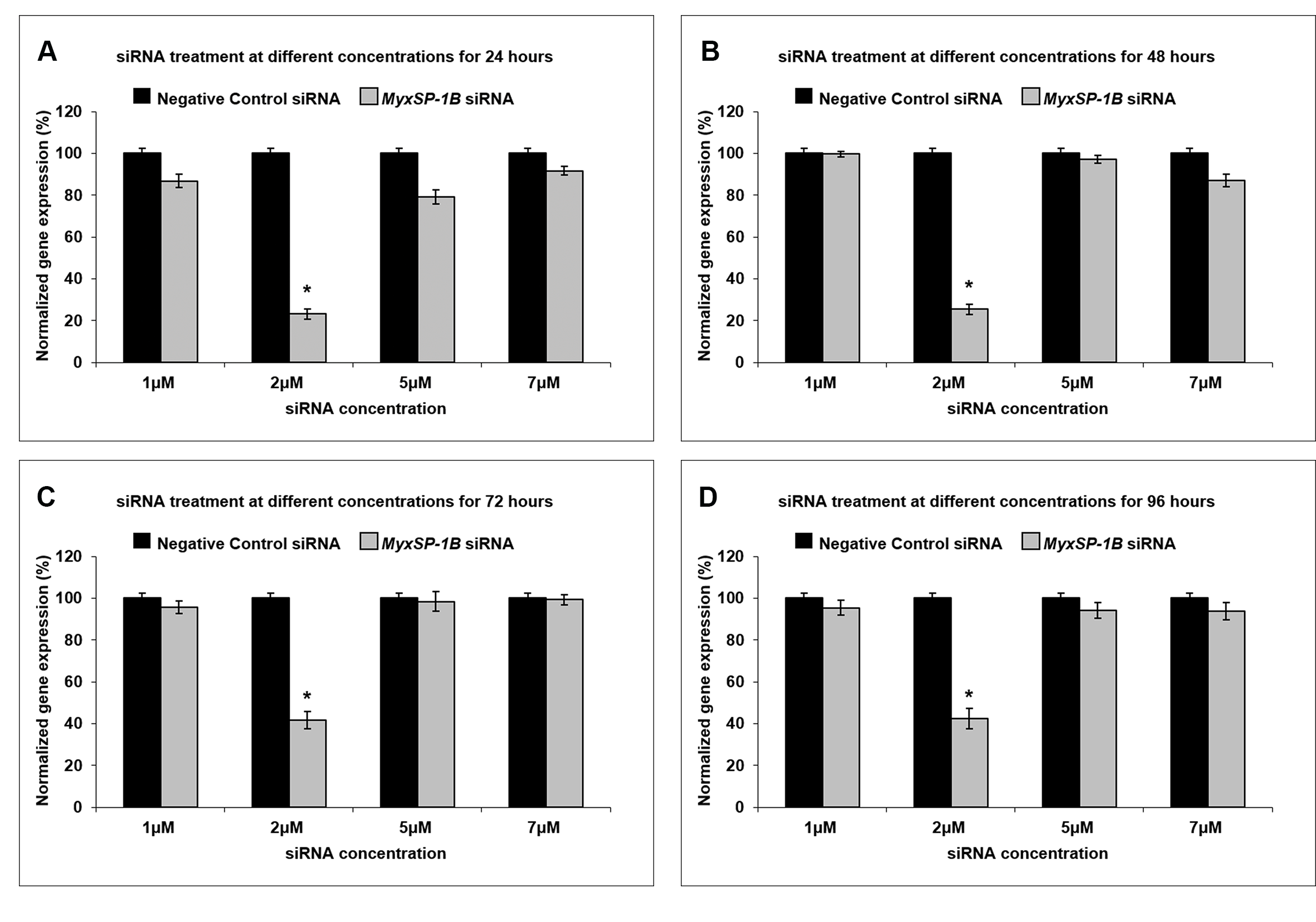

Supplement: S1 Fig — 1600 SPF T. tubifex were collected and then divided into 32 groups with each having 50 SPF T. tubifex. All groups of SPF T. tubifex were infected with M. cerebralis myxospores at the same time. At 3mpi, infected T. tubifex oligochaetes were treated with different concentrations of MyxSP-1B siRNA or negative control siRNA (1μM, 2μM, 5μM or 7μM, respectively) at 15°C for 24h (A; n = 6–8; +SE; *p<0.0001), 48h (B; n = 6–8; +SE; *p<0.0001), 72h (C; n = 6–8; +SE; *p<0.0001) and 96h (D; n = 6–8; +SE; *p<0.0001). Post-soaking, siRNA-treated T. tubifex were harvested and MyxSP-1 gene expression was evaluated using qPCR. MyxSP-1 gene expression was normalized to that of M. cerebralis β-actin. Data represent mean normalized expression +SE. Abbreviations: SPF = specific-pathogen-free; mpi = months post-infection; qPCR = real-time quantitative PCR. (TIF) [file pone.0178687.s001.tif]

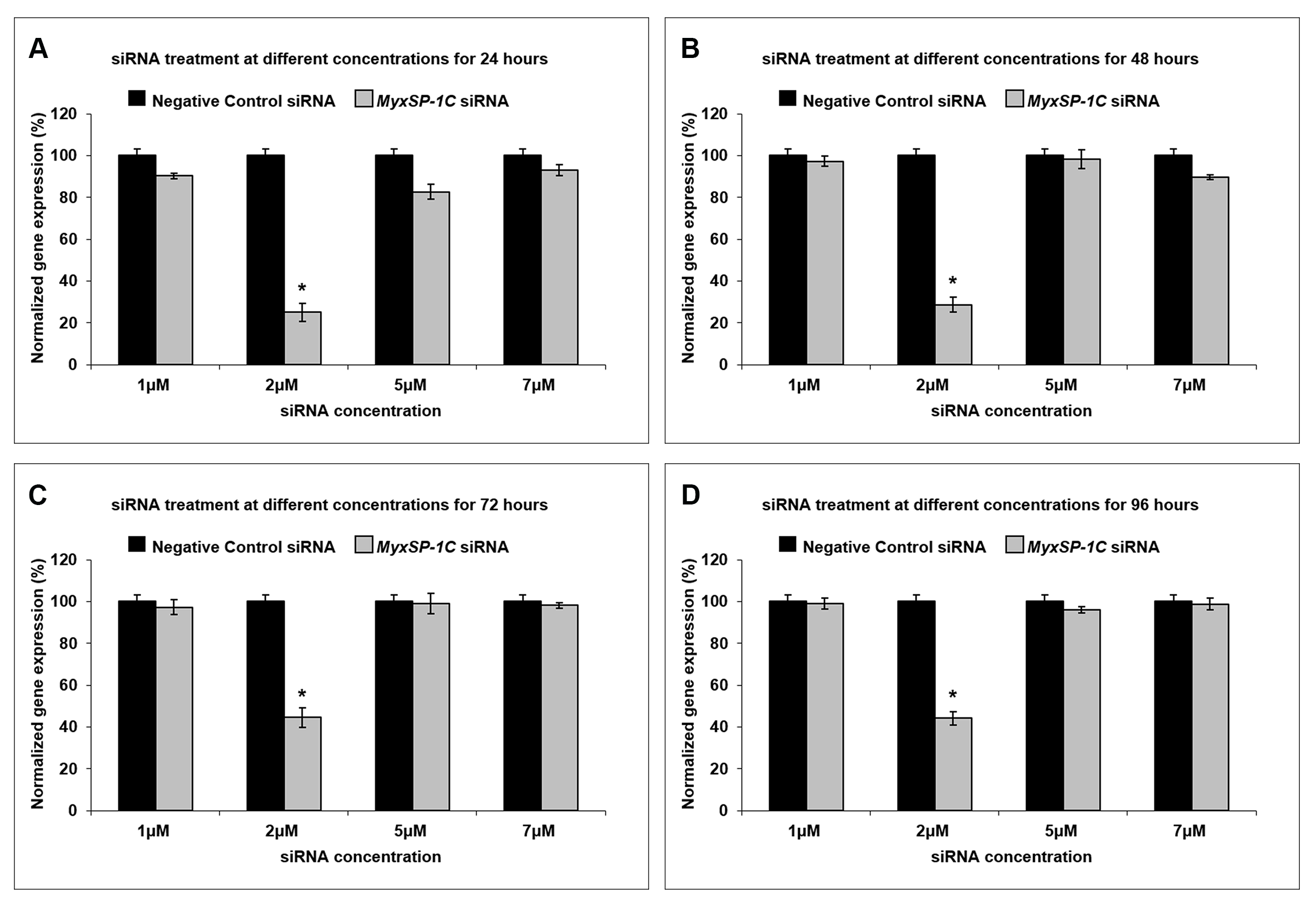

Supplement: S2 Fig — 1600 SPF T. tubifex were collected and then divided into 32 groups with each having 50 SPF T. tubifex. All groups of SPF T. tubifex were infected with M. cerebralis myxospores at the same time. At 3mpi, infected T. tubifex oligochaetes were treated with different concentrations of MyxSP-1C siRNA or negative control siRNA (1μM, 2μM, 5μM or 7μM, respectively) at 15°C for 24h (A; n = 6–8; +SE; *p<0.0001), 48h (B; n = 6–8; +SE; *p<0.0001), 72h (C; n = 6–8; +SE; *p<0.0001) and 96h (D; n = 6–8; +SE; *p<0.0001). Post-soaking, siRNA-treated T. tubifex were harvested and MyxSP-1 gene expression was evaluated using qPCR. MyxSP-1 gene expression was normalized to that of M. cerebralis β-actin. Data represent mean normalized expression +SE. Abbreviations: SPF = specific-pathogen-free; mpi = months post-infection; qPCR = real-time quantitative PCR. (TIF) [file pone.0178687.s002.tif]
