# Supplementary material for: A RNAi-based therapeutic proof of concept targets salmonid whirling disease in vivo
Source: PLoS One. 2017 Jun 2;12(6):e0178687. doi: 10.1371/journal.pone.0178687 (PMC5456292; doi:10.1371/journal.pone.0178687)
Supplement: S1 Table — 1600 SPF T. tubifex were collected and then divided into 32 groups with each having 50 SPF T. tubifex as indicated below. All groups of SPF T. tubifex were infected with M. cerebralis myxospores at the same time. At 3mpi, infected T. tubifex oligochaetes were treated with different concentrations of MyxSP-1B siRNA or negative control siRNA (1μM, 2μM, 5μM or 7μM) at 15°C for 24h, 48h, 72h and 96h, respectively. Post-soaking, siRNA-treated T. tubifex were harvested and MyxSP-1 gene expression was evaluated using qPCR. MyxSP-1 gene expression was normalized to that of M. cerebralis β-actin. Data represent mean normalized expression (n = 6–8; +SE). Abbreviations: SPF = specific-pathogen-free; mpi = months post-infection; qPCR = real-time quantitative PCR. (DOCX) [file pone.0178687.s003.docx]

**S1 Table. Effective dose for *MyxSP-1B* siRNA treatment of *M. cerebralis*-infected *T. tubifex* oligochaetes at period of peak release of TAMs.** 1600 SPF *T. tubifex* were collected and then divided into 32 groups with each having 50 SPF *T. tubifex* as indicated below. All groups of SPF *T. tubifex* were infected with *M. cerebralis* myxospores at the same time. At 3mpi, infected *T. tubifex* oligochaetes were treated with different concentrations of *MyxSP-1B* siRNA or negative control siRNA (1μM, 2μM, 5μM or 7μM) at 15˚C for 24h, 48h, 72h and 96h, respectively. Post-soaking, siRNA-treated *T. tubifex* were harvested and *MyxSP-1* gene expression was evaluated using qPCR. *MyxSP-1* gene expression was normalized to that of *M. cerebralis* *β-actin*. Data represent mean normalized expression (n=6-8; +SE). Abbreviations: SPF = specific-pathogen-free; mpi = months post-infection; qPCR = real-time quantitative PCR

| **siRNAs** | **siRNA concentration**  **(μM)** | **Duration of siRNA treatment (h)** | **Number of *T. tubifex* treated with siRNAs (n)** | **Normalized *MyxSP-1* gene expression (% ± S.E.)** | **%**  ***MyxSP-1* knockdown** |
| --- | --- | --- | --- | --- | --- |
| Negative Control siRNA | 1 | 24 | 50 | 100 ± 2.39 | 13.16 |
| *MyxSP-1B* siRNA | 1 | 24 | 50 | 86.84 ± 3.08 |  |
| Negative Control siRNA | 2 | 24 | 50 | 100 ± 2.39 | 76.76 |
| *MyxSP-1B* siRNA | 2 | 24 | 50 | 23.24 ± 2.56 |  |
| Negative Control siRNA | 5 | 24 | 50 | 100 ± 2.39 | 20.9 |
| *MyxSP-1B* siRNA | 5 | 24 | 50 | 79.10 ± 3.46 |  |
| Negative Control siRNA | 7 | 24 | 50 | 100 ± 2.39 | 8.32 |
| *MyxSP-1B* siRNA | 7 | 24 | 50 | 91.68 ± 2.10 |  |
| Negative Control siRNA | 1 | 48 | 50 | 100 ± 2.39 | 0.37 |
| *MyxSP-1B* siRNA | 1 | 48 | 50 | 99.63 ± 1.15 |  |
| Negative Control siRNA | 2 | 48 | 50 | 100 ± 2.39 | 74.54 |
| *MyxSP-1B* siRNA | 2 | 48 | 50 | 25.46 ± 2.41 |  |
| Negative Control siRNA | 5 | 48 | 50 | 100 ± 2.39 | 2.85 |
| *MyxSP-1B* siRNA | 5 | 48 | 50 | 97.15 ± 1.84 |  |
| Negative Control siRNA | 7 | 48 | 50 | 100 ± 2.39 | 12.94 |
| *MyxSP-1B* siRNA | 7 | 48 | 50 | 87.06 ± 3.12 |  |
| Negative Control siRNA | 1 | 72 | 50 | 100 ± 2.39 | 4.47 |
| *MyxSP-1B* siRNA | 1 | 72 | 50 | 95.53 ± 2.96 |  |
| Negative Control siRNA | 2 | 72 | 50 | 100 ± 2.39 | 58.38 |
| *MyxSP-1B* siRNA | 2 | 72 | 50 | 41.62 ± 4.15 |  |
| Negative Control siRNA | 5 | 72 | 50 | 100 ± 2.39 | 1.61 |
| *MyxSP-1B* siRNA | 5 | 72 | 50 | 98.39 ± 4.62 |  |
| Negative Control siRNA | 7 | 72 | 50 | 100 ± 2.39 | 0.72 |
| *MyxSP-1B* siRNA | 7 | 72 | 50 | 99.28 ± 2.51 |  |
| Negative Control siRNA | 1 | 96 | 50 | 100 ± 2.39 | 4.57 |
| *MyxSP-1B* siRNA | 1 | 96 | 50 | 95.43 ± 3.59 |  |
| Negative Control siRNA | 2 | 96 | 50 | 100 ± 2.39 | 57.4 |
| *MyxSP-1B* siRNA | 2 | 96 | 50 | 42.60 ± 4.87 |  |
| Negative Control siRNA | 5 | 96 | 50 | 100 ± 2.39 | 5.77 |
| *MyxSP-1B* siRNA | 5 | 96 | 50 | 94.23 ± 3.75 |  |
| Negative Control siRNA | 7 | 96 | 50 | 100 ± 2.39 | 6.35 |
| *MyxSP-1B* siRNA | 7 | 96 | 50 | 93.65 ± 4.13 |  |
